# A Case of Self-Induced Hydrostatic Colonic Perforation

**DOI:** 10.5339/qmj.2021.14

**Published:** 2021-04-29

**Authors:** Hassan Ali Alzahrani

**Affiliations:** Department of Surgery, Faculty of Medicine, King Khalid University, P.O. Box 641, Abha 61421, Saudi Arabia E-mail: hahassan@kku.edu.sa

**Keywords:** perforation, intestinal, pressure, hydrostatic

## Abstract

Introduction: Constipation is a common complaint. The elderly are five times more prone to constipation than young people because of the effects of medication, immobility, and a blunted urge to defecate. Many of these patients are demented, have cognitive deficits, or suffer from a psychiatric disorder.

Colonic perforation caused by hydrostatic pressure is rare, and this typically occurs during fluoroscopic studies resulting when there is excessive intraluminal colonic pressure. Self-induced colonic perforation is even rarer.

Case report: We report the case of a 95-year-old man who presented to the emergency department with an acute surgical abdomen and symptoms of sepsis. He had a history of longstanding constipation. He gave a history of frequent insertion of a rubber hose into his anal canal to relieve his constipation while rinsing his anus after defecation. After resuscitation, an emergency operation was performed, and sigmoid colon perforation was found in addition to pre-existing diverticular disease. Hartmann’s procedure was performed after abdominal washout. Unfortunately, the patient died of multi-organ failure two days after surgery.

Conclusion: Self-induced hydrostatic colonic perforation is rare. The consequence is fatal, especially in the elderly or in cases of delayed presentation.

## Introduction

Colonic perforation caused by hydrostatic pressure is rare, and it typically occurs during fluoroscopic studies or colonoscopy (barotrauma) due to excessive intraluminal colonic pressure. Self-induced hydrostatic pressure injury of the colon, on the other hand, is extremely rare. Many of these patients are demented, have cognitive deficits, or suffer from a psychiatric disorder.^1–3^

Here, we report a very rare case of a patient who developed sigmoid colon perforation resulting in fecal peritonitis and multi-organ failure due to self-induced hydrostatic water irrigation.

## Case Report

The case was of a 95-year-old man with a history of hypertension and dyslipidemia who was on hydrochlorothiazide and simvastatin. He presented to our hospital with abdominal pain and frequent vomiting. The patient was suffering from longstanding constipation. He used to habitually insert a rubber hose into his anal canal and use a hydrostatic water tap to relieve his constipation. He gave a history of performing this habit 24 hours before his presentation.

The patient was distressed with a temperature of 36.5°C, a respiratory rate of 30 per minute, a heart rate of 130 per minute, and blood pressure of 130/65 mmHg on physical examinations. Abdominal examination revealed a tender, non-distended abdomen with guarding allover. A soft stool with an enlarged, firm prostate was revealed during a digital rectal examination.

**Figure 1.**
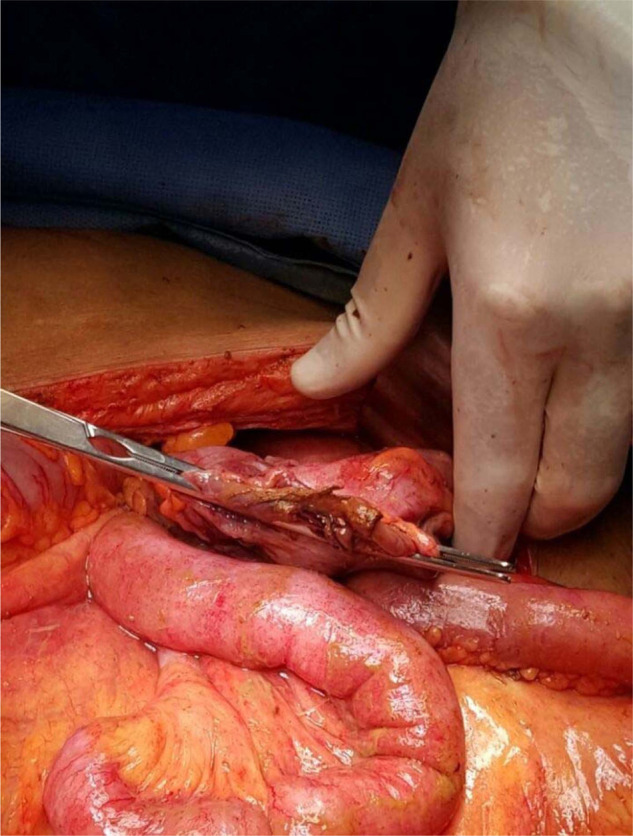
Supine abdominal X-ray with high suspicion of free air under the diaphragm

Intravenous fluid, analgesia, and broad-spectrum antibiotics were used to rapidly resuscitate the patient. The patient could not tolerate standing for an upright chest X-ray, and a supine abdominal X-ray showed a suspicion of free air (Figure 1).

He was found to have a low white blood cell count of 1.5 × 10^3^/μ L, a hemoglobin level of 10.2 g/L, a platelet count of 194 × 10^9^/L, and an international normalized ratio of 1.4.

The diagnosis of bowel perforation with fecal peritonitis was made. Various causes were considered including stercoral perforation. However, based on the clinical context, self-induced hydrostatic colonic irrigation was the most likely cause of perforation.

**Figure 2.**
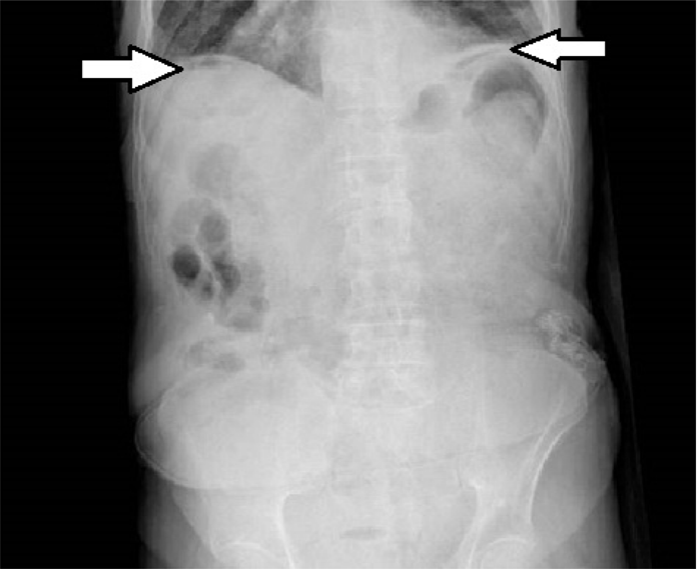
Intraoperative image of perforation controlled with bowel clamp

The patient was stratified as an ASA IV and taken to the operating room at high risk of death and perioperative complications. An emergency midline laparotomy was performed, which revealed fecal matter all over the abdomen and a 2-cm free perforation of the sigmoid colon in addition to multiple diverticula in which some of them were included in the tear (Figure 2).

Abdominal washout and Hartmann’s procedure were carried out. He was shifted to the intensive care unit for critical continuous monitoring. Unfortunately, the patient died of multi-organ failure two days after surgery.

Pathological examination of the resected sigmoid colon confirmed the presence of sigmoid diverticula with a 2-cm tear.

## Discussion

Perforations caused by self-induced hydrostatic pressure are exceptionally rare, with few cases described in the literature.^2,3^ Here, we summarize and compare these reported cases in terms of clinical presentation, management, and outcome (Table 1). The age and the time prior to presentation were the main differentiating factors between our case and the reported cases and would most likely be attributed to the adverse outcome.

Fleet enema is a relatively invasive method to treat constipation. This method is used to relieve constipation refractory to treatment with oral and suppository forms in clinical settings.^4^ Most individuals with constipation treat themselves without seeking medical advice, usually with over-the-counter medications; however, a few of them might carelessly use household items to simulate what they have usually received in healthcare facilities.

Colorectal perforations secondary to mechanical (puncture injuries) or hydrostatic pressure are well-known complications of certain clinical procedures, such as colonoscopy and barium enema.^5,6^ Review of literature by Khan et al., reported iatrogenic perforations rates of 0.06% with colonoscopies, and approximately 0.02% to 0.24% with barium enema. Iatrogenic perforations were found to have more favorable outcomes due to early diagnosis and active management.^7^

Apart from many reported cases of perforation in patients who underwent fleet enema, Handley et al., reported three cases of rectal perforations due to colonic irrigation administered outside the clinical setting by alternative medicine practitioners.^8^ Most of these cases were related to the device tip;^4^ other causes were related to localized weakness of the rectal wall, obstruction, or the position of the patient when the enema was performed.^9,10^ It has been found that manual squeezing of the barium enema bag can produce an intraluminal pressure of 150 mmHg, whereas perforation can result from pressures in the range of 50 to 109 mmHg.^11,12^

In recent years, transanal irrigation has become a well-established treatment for chronic constipation and fecal incontinence in selected patients.^8,13^ The risk of perforation with transanal irrigation is estimated to be 20 per 1 million procedures.^14^ A recent study showed the increased risk of perforation in patients with a fragile bowel wall due to scarring from prior rectal surgery or even a rectal anastomosis, irradiation, or diverticular disease. Diverticular disease was the third greatest contributing factor to perforation in the study, while the number one contributing factor was previous pelvic organ surgery.^15^

The most susceptible location of hydrostatic pressure-induced perforation is the rectosigmoid colon, due to the S-shaped distal large bowel configuration, with the lateral supports of the rectum, making the area of rectosigmoid the first part of the colon to be struck by hydrostatic pressure.^16,17^ Most similar perforations (enema-induced ones) were reported at the antimesenteric border of the sigmoid colon with fecal peritonitis, as seen in our case.^9^

Rapid resuscitation, intravenous antibiotics, resection of the tear, peritoneal washout, and diverting colostomy are necessary. Severe life-threatening peritonitis and endotoxemia may result if fecal matter escape into the peritoneal cavity.^18^ Despite the outcome variations internationally, emergency abdominal surgery is life-threatening with an overall thirty-day mortality of 5.4%; therefore, fatal cases should be expected, particularly in presence of hydrostatic pressure that results in widespread fecal contamination as observed in our case.^19^

## Conclusion

Colonic perforation due to self-induced hydrostatic pressure is extremely rare, with few reported cases in the literature. Patients with constipation should be educated about the risk of serious complications, including death, from injudicious use of hydrostatic colonic irrigation.

## Figures and Tables

**Table 1:** List of reported cases of colonic perforation due to self-induced hydrostatic pressure in the literature

No	The Journal	Age	Presentation (clinical, radiological and biochemical)	Time before presentation	Operative findings	Procedure performed
1	Ann Surg 1935;102: 471-2.	60	• Abdominal pain, board-like rigidity, absence of bowel sound. • Temperature of 35.8°C, blood pressure of 140/80, pulse rate of 80 and respiratory rate of 30. • White blood cell count of 10 × 10^3^/**μ** L.	Not specified	A longitudinal 3-cm tear at the apex of sigmoid flexure anteriorly	Primary repair in a transverse fashion
2	J Emer Med 2013;44: 344-348.	61	• Abdominal pain, rectal bleeding, distended abdomen, no signs of peritonitis. • Temperature of 37.2°C, blood pressure of 150/80, pulse rate of 97 and respiratory rate of 22. • Abdominal X-ray showed dilated small bowel loops. CT showed mesenteric air bubbles of the rectosigmoid colon. • White blood cell count of 11 × 10^3^/**μ** L.	6 hours	A 5-cm mesenteric hematoma, with a 3-cm tear in the mesenteric border of the colonic wall	Hartmann’s procedure
3	J Emer Med 2013;44: 344-348.	45	• Severe lower abdominal pain, tender over the lower abdomen, without signs of peritoneal irritation. • Temperature of 38.1°C, blood pressure of 140/80 mmHg, pulse rate of 92 beats/minute, and respiratory rate of 24 breaths/minute. • Abdominal X-ray was unremarkable. CT scan showed a defect in the wall of the distal sigmoid colon, with spillage of fecal material into the mesentery. • White blood cell count of 10 × 10^3^/**μ** L.	2 hours	A 2-cm tear in the colonic wall along the mesenteric border	Primary resection and anastomosis
